# Brain markers predicting response to cognitive‐behavioral therapy for social anxiety disorder: an independent replication of Whitfield-Gabrieli et al. 2015

**DOI:** 10.1038/s41398-021-01366-y

**Published:** 2021-05-01

**Authors:** Yoni K. Ashar, Joseph Clark, Faith M. Gunning, Philippe Goldin, James J. Gross, Tor D. Wager

**Affiliations:** 1 Department of Psychiatry, Weill Cornell Medicine, New York, NY USA; 2 Department of Psychology and Neuroscience, University of Colorado Boulder, Boulder, CO USA; 3 Betty Irene Moore School of Nursing, University of California, Davis, Davis, CA USA; 4 Department of Psychology, Stanford University, Palo Alto, CA USA; 5 Psychological and Brain Sciences Department, Dartmouth College, Hanover, NH USA

**Keywords:** Predictive markers, Human behaviour

## Abstract

Predictive brain markers promise a number of important scientific, clinical, and societal applications. Over 600 predictive brain markers have been described in published reports, but very few have been tested in independent replication attempts. Here, we conducted an independent replication of a previously published marker predicting treatment response to cognitive-behavioral therapy for social anxiety disorder from patterns of resting-state fMRI amygdala connectivity^1^. The replication attempt was conducted in an existing dataset similar to the dataset used in the original report, by a team of independent investigators in consultation with the original authors. The precise model described in the original report positively predicted treatment outcomes in the replication dataset, but with marginal statistical significance, permutation test *p* = 0.1. The effect size was substantially smaller in the replication dataset, with the model explaining 2% of the variance in treatment outcomes, as compared to 21% in the original report. Several lines of evidence, including the current replication attempt, suggest that features of amygdala function or structure may be able to predict treatment response in anxiety disorders. However, predictive models that explain a substantial amount of variance in independent datasets will be needed for scientific and clinical applications.

Predictive brain markers promise a number of important scientific, clinical, and societal applications^2^. Yet, success in this domain will depend on the replicability and generalizability of brain marker predictions^3–6^. Replicability is at the foundation of scientific enterprise, and it has recently become a subject of increased attention in a number of fields including psychology^7,8^, translational neuroscience^9,10^, medicine^11,12^, and more^13–16^. This focus has generated a growing awareness that many published findings cannot be replicated, along with a move towards methods promoting replicability^17–21^.

The replicability of most brain markers has not been assessed. A recent review found that of ~450 published predictive brain markers, only ~10% have been tested on independent data, and only two clinical markers (one for Alzheimer’s disease and one for Parkinson’s disease) have been subjected to broader tests of generalizability^4^. More independent replication attempts of brain markers are needed, both to assess the current state of replicability and to spur the development of replicable predictive models.

Here, we conducted an independent replication of a previous report that response to cognitive-behavioral therapy (CBT) for social anxiety disorder (SAD) could be accurately predicted from baseline brain connectivity^1^. The authors reported that baseline amygdala-seeded functional connectivity explained 21% of the variance in treatment response, with incremental validity above and beyond a paper-and-pencil measure of baseline symptom severity. Treatment response was predicted by a linear combination of positive amygdala connectivity with a subgenual cingulate/caudate/putamen cluster, negative amygdala connectivity with bilateral central sulcus clusters, and negative amygdala connectivity with a right temporal-occipital cluster. We conducted our replication attempt in an existing dataset^22,23^ which was similar to the original study in key respects, with baseline resting-state fMRI collected on SAD patients prior to CBT treatment, though some differences in sample characteristics and treatment implementations are noted, described further below.

Alterations in amygdala function and structure have been one of the most reliable findings that distinguish SAD patients from healthy controls, including greater amygdala responses to threatening social stimuli and altered profiles of resting connectivity^24,25^. Several reports have also highlighted functional or structural properties of the amygdala that are impacted by CBT, often correlating with improvements in clinical outcomes^22,26–28^. And, paralleling the Whitfield-Gabrielli et al. findings studied here, two other studies have reported that different features of amygdala function predict SAD treatment response to CBT^29,30^. Yet, to our knowledge, none of these studies have been directly subjected to independent replication attempts. Collectively, this has generated an important set of findings with a coherent, consistent focus on the amygdala—but with unknown replicability.

Independent replication attempts of precisely specified models are needed to advance the field toward clinical and societal applications. Scientific collaborations will support this effort, leading to a cumulative science creating research products with both clinical and scientific applications. Here, we test the precise model specified in the original report. In addition, we also tested a variant of the original model, to better understand how amygdala connectivity predicts treatment response in the replication dataset.

## Methods

The replication and original datasets were similar in key respects, with a comparison provided in Table 1 and discussed in greater detail below. The replication dataset has been previously described in studies examining the effects of cognitive-behavioral therapy (CBT) on brain function (NCT00380731)^22^. No analyses aiming to predict treatment response from baseline imaging have been previously reported in the replication dataset.

**Table 1:** Comparison of original and replication datasets.

	Original report	Replication dataset
Primary reference	Whitfield-Gabrieli et al.^1^	Goldin et al., 2012, 2013
Patient population	Social anxiety disorder	Social anxiety disorder
Sample size	38	42
Age, years	*M* = 29.2, range 18–49	*M* = 34.5, SD = 8.89, range 21–53
Gender	63% male	45.2% male
Race/Ethnicity	Not reported	54.8% Caucasian, 26.2% Asian, 7.2% Filipino/Pacific Islander, 7.1% Hispanic/Latinx, 2.4% Black, 2.4% more than one
Education, years	Not reported	*M* = 17.0, SD = 2.20, range 13–21
Treatment	12 weeks of group CBT	16 weeks of individual CBT
SAD definition and inclusion criteria	SAD diagnosis confirmed using either the SCID or ADIS for DSM-IV; LSAS ≥60	SAD diagnosis confirmed using the ADIS for DSM-IV, with ≥4 ADIS clinical severity rating; LSAS≥60
Primary clinical outcome	LSAS	LSAS (baseline *M* = 82.0 [SD = 17.9]; post-CBT *M* = 51.3 [24.2])
Medication status	Unmedicated for at least 2 weeks prior to baseline fMRI	Unmedicated for at least 1 year prior to baseline fMRI
Psychiatric comorbidities	Other mood or anxiety disorders permitted if SAD judged to be the predominant disorder; other psychiatric conditions excluded.	GAD, agoraphobia, specific phobia, panic disorder, and dysthymia permitted; other psychiatric conditions excluded. 11.9% with current Axis I comorbidity; 26.2% with past Axis I diagnosis.
Age of SAD onset, mean (SD), years	12.2	14.26 (8.32)
SAD duration, mean (SD), years	17.4	20.45 (12.91)
Scanner	3 T Siemens Trio Tim	GE 3-T Signa
Headcoil	Siemens 32-channel	Quadrature coil
Scan parameters	6 min, TR = 6 s, 2x2x2 mm resolution, gradient echo	5 min, TR = 1.5 s, 3.4 ×3.4 ×4.5 mm resolution, spin echo
Task	Fixate on crosshairs	Fixate on crosshairs
Recruitment region	Boston area	San Francisco Bay Area

*CBT* cognitive behavioral therapy, *LSAS* Liebowitz Social Anxiety Scale, *GAD* generalized anxiety disorder, *SAD* social anxiety disorder.

### Participants

Participants in the replication dataset were recruited through referrals and web listings between 2007 and 2010. Participants were required to have a principal diagnosis of a SAD, with no current pharmacotherapy or psychotherapy. SAD was assessed by a diagnostic interview conducted by Ph.D.-trained clinical psychologists using the Anxiety Disorders Interview Schedule for DSM-IV (ADIS- IV)^31^, which has strong inter-rater reliability^32^. Diagnostic criteria for SAD were defined as greater than moderate fear in five or more distinct social situations, LSAS score of ≥60, and a clinician-assigned ADIS-IV clinical severity rating of 4 or greater (0–8 scale) for SAD. This was similar to the original study, which used either the SCID or ADIS for DSM-IV to confirm SAD diagnosis and also required LSAS score ≥60 (Table 1).

Exclusion criteria included current or past CBT, history of neurological disorders, or meeting diagnostic criteria for any current psychiatric condition other than generalized anxiety disorder, agoraphobia without panic attacks, specific phobia, panic disorder, or dysthymia. This was similar but not identical to the original study, which allowed the presence of comorbid mood disorders if SAD was judged to be the predominant disorder.

We required participants to be unmedicated for at least 1 year prior to the baseline scan; the original study required a period of at least two weeks without psychiatric medications. We also required participants to be right-handed based on the Edinburgh Handedness Inventory^33^ and to pass an MRI safety screen. The original study did not require right-handedness (though most participants in the sample were right-handed). Participants in the replication dataset provided informed consent in compliance with the Stanford University Institutional Review Board.

### Procedures

After passing a telephone screen and an in-person eligibility session, participants completed a baseline assessment session including fMRI. Participants were then randomly assigned using biased coin randomization to either individual CBT (*n* = 38) or to a waitlist (WL) control group (*n* = 37) who were subsequently offered CBT. Pre-randomization resting-state fMRI data were collected on *n* = 53 subjects of these subjects (*n* = 30 CBT immediate, *n* = 23 CBT post-waitlist). All participants who completed at least 12 of 16 CBT sessions were included in analyses, with their last available measure of social anxiety symptom severity used in analyses (last observation carried forward).

CBT included 16 individual weekly sessions over 4 months. It was delivered using *Managing Social Anxiety: A Cognitive-Behavioral Therapy Approach*, a manualized treatment protocol that included a therapist guide^34^ and a client workbook^35^. All four study therapists had demonstrated proficiency in CBT with training cases prior to treating study patients. Therapists were trained and supervised by Richard Heimberg, PhD, an expert in CBT for SAD and one of the developers of the treatment protocol used here. All four therapists demonstrated adherence to treatment protocols, as verified by independent raters, as detailed in the original report^23^.

Participants in the WL group initiated individual CBT~19 weeks after the baseline brain scan. We included these participants in analyses as it is unknown whether the biomarker tested here predicted the effects of CBT specifically, placebo effects, the natural history of symptoms, or some combination of these factors since the original report did not include a control group. Given the likelihood that the biomarker predicted a combination of these factors^36^, we reasoned that the post-waitlist CBT participants also offered a fair test for the biomarker while allowing us a larger sample size. Thus, considering dropout, the final sample consisted of *n* = 42 (*n* = 25 CBT-immediate, *n* = 17 CBT post-waitlist). For completeness, we also conducted the replication in the CBT-immediate group only.

### Measures

The Liebowitz Social Anxiety Scale (LSAS) self-report form^37,38^ was the primary outcome, as in the original report. LSAS scores range from 0 to144, with traditional cutoffs for mild, moderate, and severe symptom severity at 30, 50, and 90. Treatment response was defined as the pre-to-post-treatment change in LSAS. The LSAS has excellent reliability and construct validity^39^, and its internal consistency was excellent in this study (Cronbach’s alpha = 0.91).

### Data acquisition

Imaging was performed on a GE 3-T Signa magnet with a T2*-weighted gradient-echo spiral-in/spiral-out pulse sequence and a custom-built quadrature “dome” elliptical birdcage head coil (GE Healthcare, Milwaukee, Wisconsin). Participants completed a 5-min resting-state functional run while fixating on cross-hair visual stimuli. 200 functional volumes were obtained from 22 sequential axial slices (repetition time = 1.5 s, echo time = 30 ms, flip angle=60°, field of view = 22 cm, matrix = 64×64, single-shot, resolution = 3.438 mm^2^ × 4.5 mm). Three-dimensional high-resolution anatomical scans were acquired using fast spin-echo spoiled gradient recall (0.85942 ×1.5 mm; field of view = 22 cm, frequency encoding = 256).

In the original report, a 6-min resting-state scan was collected on a 3 T Siemen’s Trio Tim scanner was collected while participants fixated on a cross-hair (T2* weighted gradient echo repetition time/echo time/Flip angle = 6000 ms/30 ms/90°, 67 contiguous interleaved oblique slices, voxel size: 2.0 mm^3^).

### Preprocessing of fMRI data

Preprocessing followed the same procedures and tools described in the original report, using the Conn toolbox^40^ wrapping SPM12 for preprocessing routines. We performed slice time correction, motion estimation and realignment, normalization to MNI305 space, and spatial smoothing with an 8 mm FWHM Gaussian filter. GLM regression was used to remove the influence of with the following nuisance covariates: six head motion parameters and their first-order temporal derivatives, the first three components of white matter and CSF tissue compartments, and spike regressors identifying volumes flagged as outliers by the Artifact Detection Tool^40^ (an image was defined as an outlier if the head displacement in any direction was ≥ 0.5 mm from the previous frame, or if the global mean image intensity ≥ 3 standard deviations above the mean image intensity for the scan). The resulting residual BOLD time-series were then band-pass filtered (0.01 Hz < f < 0.10 Hz). The replication dataset had acceptable levels of head motion, quantified as framewise displacement (FD) (*M*_FD_ = 0.13 mm, Median_FD_ = 0.06 mm, SD_FD_ = 0.13 mm).

### Comparison of the original and replication datasets

The original and replication datasets were similar in key respects. They also differed in some characteristics, such as group vs. individual CBT, scan acquisition parameters, the geographic region from which patients were recruited, and other characteristics (see Table 1 for a full comparison).

In addition, the original study randomized subjects to receive either D-cycloserine or placebo in conjunction with CBT. No drug vs. placebo differences emerged, and the authors collapsed across conditions in their analyses. The treatment in the original study might thus be considered “placebo-enhanced” CBT. While meta-analyses show a placebo effect of *g* = 0.39 in SAD^41^, it is unknown to what extent this effect is additive, interacting, or fully overlapping with psychotherapy effects^36^, and so it cannot be determined to what extent drug administration impacted outcomes in the original study. Importantly, randomization to D-cycloserine or placebo occurred after the baseline imaging session in the original study, so there were no drug effects on functional connectivity. No medication was administered in the replication study, a potentially important difference between the two datasets.

### Model specification

Details regarding the predictive model specification and the cluster masks were provided in personal communications with the original authors. Mean connectivity between the amygdala and the single positive connectivity cluster was averaged with mean amygdala connectivity with the three negative connectivity clusters. This formed a single connectivity term, which was Fisher-transformed and Z-scored across subjects. This connectivity term was then submitted to a GLM along with baseline symptom severity (LSAS) to predict treatment response, operationalized as the pre-to-post-treatment change in LSAS (ΔLSAS). The final predictive model derived in the original report was:

ΔLSAS = 0.6194 * baseline_LSAS + 8.6290 * amyg_conn − 9.9763 (1), where *amyg_conn* refers to the amygdala connectivity term. The original report compared predictions from (1) to a compact model including only baseline LSAS and an intercept term as predictors.

Precisely specified models are necessary for a replicable, cumulative science. We thus sought to apply this exact model to our data. We made one modification, dropping the intercept term from the model (and mean-centering all model variables in the replication dataset). This removed intercept effects in treatment response, as these are reasonably expected to vary from study to study.

The original model included dependence between a predictor (baseline LSAS) and the outcome (change in LSAS from baseline to post-treatment). Thus, we first tested whether the covariance between baseline and change in LSAS was similar across the original and replication datasets. We compared model predictions derived from the optimal OLS model fit in the replication data to predictions using the parameter estimate from the original model (i.e., β_baseline_LSAS_ = 0.6194).

In addition, to explore a more flexible form of replication, we conducted a GLM estimating new parameter estimates for the original model terms. Here, we tested whether the amygdala connectivity term significantly predicted treatment response, controlling for baseline LSAS.

The original report also reported successful prediction of treatment response from multivoxel pattern analyses (MVPA) of resting fMRI and from diffusion tensor imaging (DTI). We were unable to apply the MVPA-derived model to our data: One of the predictive clusters in the MVPA model was an inferior cerebellar region for which we had incomplete coverage in the replication dataset. Similarly, DTI was not collected in the replication dataset.

### Model assessment

We assessed performance by comparing predictions from the full model to a compact model including only the baseline LSAS term. Prediction performance was measured using two metrics, following recent recommendations^3,42^.

First, we computed an absolute measure of improvement in prediction, normalized mean square error (NMSE). We computed the mean squared error (MSE) between the observed data and the predictions from the full model, divided by MSE between the observed data and the predictions from the compact model. We then computed “prediction *R*^2^”, defined as 1 – NMSE, providing the proportional reduction in error for the full model vs. the compact model^42^.

The second metric we used was a model-based *R*^2^. We computed the squared Pearson correlation between the observed data and the full model predictions, as compared to the squared correlation between the observed data and the compact model predictions. Correlation provides a scale-free measure of predictive accuracy, which may be appropriate for testing the model in independent data, though in doing so it provides increased model flexibility. Squaring the correlation coefficient provided a model-based estimate of *R*^2^.

Statistical significance of prediction and model-based *R*^2^ was assessed by repeatedly permuting the amygdala connectivity term across subjects, generating a null distribution, and comparing the unpermuted result to the 95th percentile of the null distribution (10,000 permutations). Analyses were conducted using the CanlabCore toolbox, a freely available MATLAB© toolbox for flexible neuroimaging analyses: https://github.com/canlab/CanlabCore. Data and code for analyses are publicly available at https://github.com/yonestar/WhitfieldGabrieli2015_replication.

## Results

### Predicting treatment response from baseline symptoms

In the original report, the model including only baseline social anxiety symptom scores (“compact model”) predicted 12% of the variance in pre-to-post-treatment change scores: Higher pre-treatment scores predicted greater pre-to-post-treatment symptom reductions, owing both to regression to the mean and to dependence between the two variables.

In the replication dataset, baseline social anxiety symptoms explained 20% of the variance in pre-to-post-treatment change scores. The OLS parameter estimate for baseline symptom scores in the replication dataset was β_baseline_LSAS_ = 0.66, numerically similar to the parameter estimate from the original model (β_baseline_LSAS_ = 0.62). We confirmed that the OLS parameter estimate provided only a very small improvement in prediction over the original parameter estimate (<1%, prediction *R*^2^ = 0.0012). Since both the OLS-derived and the original parameter estimate for baseline social anxiety symptoms performed very similarly in predicting treatment response in the replication data, we used the original parameter estimate in the replication analyses to provide the most direct test of model replication.

### Predicting treatment response from amygdala connectivity

**Fig. 1:**
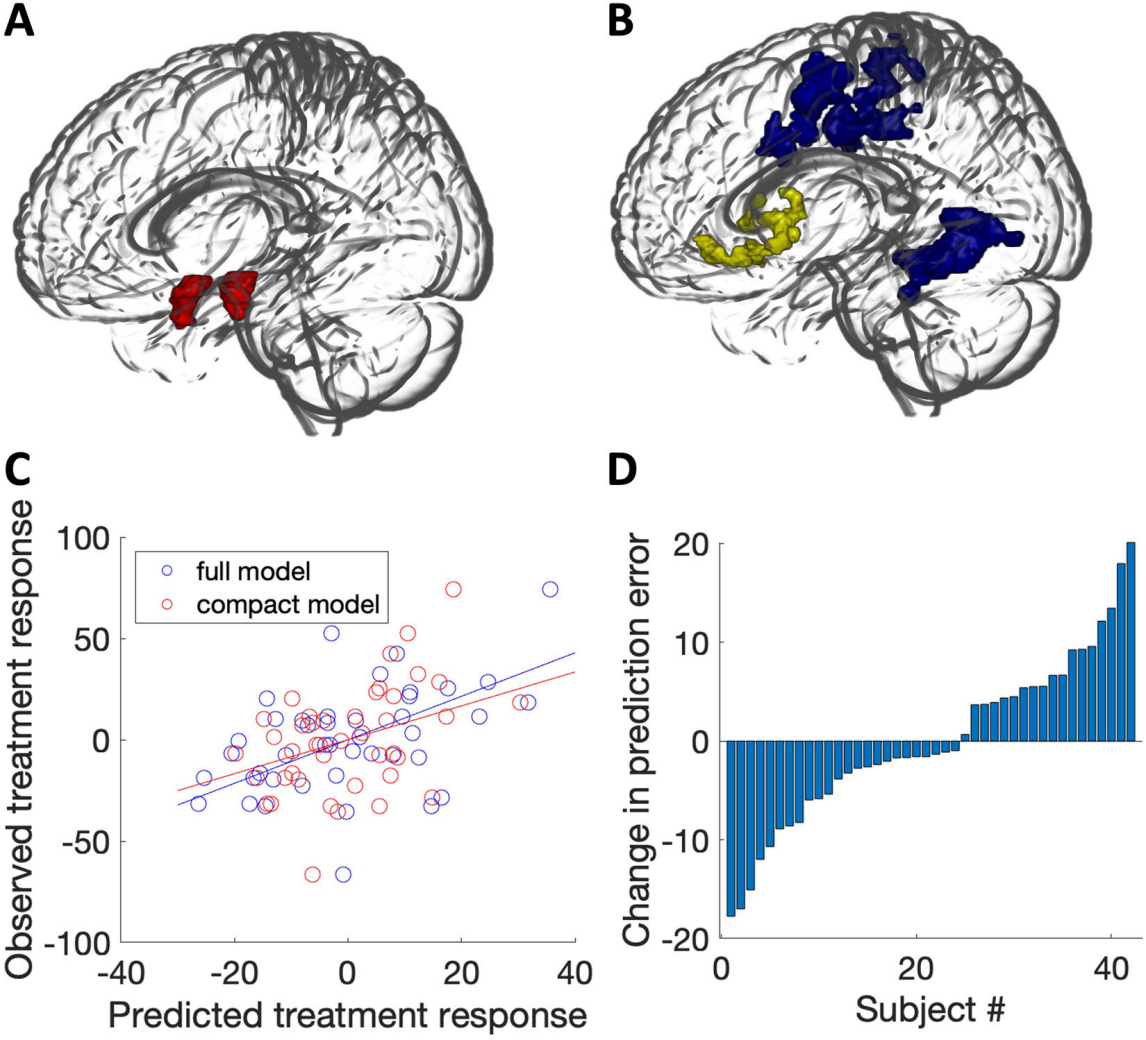
The predictive model and independent replication results. **A** The seed region for the connectivity-based predictive model: bilateral, anatomically defined amygdala (red). **B** Treatment response was predicted by a linear combination of greater amygdala connectivity with a subgenual cingulate/caudate/putamen cluster (yellow) and reduced amygdala connectivity with bilateral central sulcus clusters and right temporal-occipital clusters (blue). **C** Predicted treatment response for the compact model (including only baseline symptom severity, red points) vs. the full model (also including amygdala connectivity, blue points). Successful replication would be indicated by an improved prediction for the full model vs. the compact model. **D** Changes in prediction errors for the full vs. compact model for each subject. The inclusion of amygdala connectivity slightly improved model predictions (increase of 2% variance explained), with marginal statistical significance. Brain images were created using MRICroGL.

In the original report, the addition of the amygdala connectivity term led to a total 33% variance explained, a substantial increase of 21% over the variance explained by baseline social anxiety symptoms alone. In the replication dataset, the amygdala connectivity term explained an additional 2% of the variance (Fig. 1C; prediction *R*^2^ = 0.016, model-based *R*^2^ = 0.020). This improvement in prediction was marginally significant, model-based *p* = 0.097 and prediction *R*^2^ *p* = 0.101, rendering it unclear whether the small increase in predictive power afforded by amygdala connectivity was due to chance.

Visual inspection of the data confirmed that findings were not driven by outliers (Fig. 1D). We confirmed that all three terms in the model were approximately normally distributed, Anderson-Darling test, *p*s > 0.4.

### Predicting treatment response with increased model flexibility

To provide a more flexible test of model replication, we estimated a GLM including the same model terms as in the original report. Controlling for baseline LSAS, the amygdala connectivity term did not significantly predict treatment response, β_amyg_conn_ = 4.78, *t*(40) = 1.31, *p* = 0.20.

### Testing model predictions in CBT-immediate subset

In the subset of 25 participants who received CBT immediately after the baseline neuroimaging session (CBT-immediate), the model did not predict treatment response. In this subset, predictions from baseline LSAS alone (compact model), the OLS estimate for β_baseline_LSAS_ = 0.65, highly similar to original model value (β_baseline_LSAS_ = 0.62). We thus used the original model value for β_baseline_LSAS_, as with the full sample attempt. Relative to the compact model, the full model including amygdala connectivity did not improve the prediction of treatment response, prediction *R*^2^ = −0.019, *p* = 0.18, model-based *R*^2^ = 0.007, *p* = 0.20. Fitting the predictive model in this subset of participants, the amygdala connectivity term did not significantly predict treatment response controlling for baseline symptom severity, β_amyg_conn_ = 3.84, *t*(40) = 0.81, *p* = 0.43.

## Discussion

We conducted an independent replication of a model previously shown to predict response to CBT for SAD from resting-state amygdala-seeded fMRI connectivity^1^. We tested the precise model developed in the original report. This model provided a small improvement in the prediction of treatment response. It explained an additional 2% of the variance beyond baseline symptom severity, which attained marginal statistical significance and was approximately 1/10th of the effect size in the original report. A more flexible test of model replication, which used the amygdala connectivity target regions from the original report and estimated model parameters on the replication dataset, found no significant prediction of treatment response. Overall, our results support the hypothesis that some features of the amygdala might predict treatment response in SAD, but that more strongly predictive models must be developed for scientific and clinical applications.

The original and replication datasets were similar in many characteristics, and at the same time, differences between them in patient characteristics, treatment implementations, and fMRI parameters may have contributed to the current findings. The samples were similar in SAD age of onset, SAD duration, and inclusion/exclusion criteria (Table 1). And yet, different symptom profiles within a diagnostic category, as well as different patterns of psychiatric comorbidities, have been associated with functional connectivity differences^43–46^. CBT implementations also varied between the two studies, including individual vs. group CBT and the “placebo enhancement” of CBT in the original dataset, another important difference. And scanner hardware and acquisition parameters differed between the two studies. While this was not an exact replication, we believe that predictive markers will need to be robust to reasonable variations in many of these characteristics for practical scientific and clinical applications.

We focus our discussion here on five factors of interest that likely influenced the current findings and can also guide future attempts to develop generalizable predictive biomarkers. These include: (a) sample size, (b) reliability of fMRI connectivity measures, (c) dimensional rather than categorical models of dysfunctional phenotypes, (d) incremental validity and “broad” data, and (e) data sharing and multi-team collaborations.

First, both the original and replication datasets were underpowered due to relatively small sample sizes. With approximately *N* = 40 in each dataset, these studies provide only 50% power to detect a medium effect (*ρ* = 0.3). Stable estimates for correlation coefficients describing medium effects require *N* = 100–250, depending on the desired confidence interval^47^. The original findings—21% variance explained by amygdala connectivity in a sample of size *N* = 38—are accompanied by wide 95% confidence intervals ranging from 2.7 to 46%.

A second factor is the reliability of fMRI connectivity measures. In the original and replication datasets, scans were 6 and 5 min, lengths unlikely to yield reliable seed-based connectivity estimates^48,49^. Recent work with highly sampled subjects has found that 30–40 min of motion-censored data per subject provides reliable connectivity estimates^50,51^, with perhaps twice this scan length needed for subcortical structures^52^. In addition to longer scans, advances in acquisition and analysis technologies, such as multi-echo fMRI^53^ and custom head molds^54^, improve data quality. Analytic approaches can also improve reliability, with multivariate markers likely providing a broader base of support relative to single regions of interest^6,55^. In addition, methods for improved inter-subject alignment^56,57^ and systematic approaches for developing biomarker pipelines^58,59^ will improve performance. Finally, spatial resolution is an additional consideration, especially for smaller structures like the amygdala, and effective resolution will be determined by the smoothing kernel applied.

Third, both and the original and replication studies considered the SAD diagnosis as a categorical indicator of a shared dysfunction. However, it is well known that psychiatric patient populations are heterogenous: a shared diagnosis does not require shared mechanisms or even shared symptoms^60,61^. Predictive markers focused on patient subtypes or dimensional descriptions of dysfunction may relate more strongly to biology, providing greater traction for predictive biomarkers^46^. Several dimensional models have been recently proposed, including the NIH research domain criteria^62^ and the Hierarchical Taxonomy of Psychopathology^63^, and some recent studies have adopted a dimensional subtyping approach with encouraging results^43,64,65^.

A fourth factor is an incremental validity and “broad” data^66^. Biomarkers will be most useful if they have predictive utility above and beyond measures that are cheaper and easier to acquire. Demonstration of incremental validity requires collecting a “broad” set of measures from multiple channels (ecological momentary assessment, behavior, physiology, smartphone usage data, etc.) to compare with neuroimaging-based prediction. Critically, “broad” data will advance understanding of how environmental influences mediate the relationship between biology and phenotypic measures of dysfunction (e.g., biomarkers predictions may only hold in a particular socioeconomic group or only for subjects with high inflammation^67^).

Fifth, for a cumulative scientific process to yield generalizable predictive biomarkers, data sharing and collaborative replication efforts are needed, such as the current effort^4,17,19,21^. Software tools, data sharing platforms, and reproducibility pipelines are needed to support this effort^21,68,69^. We believe it is important to replicate precisely defined models^70^. For example, the amygdala is a sensible focus point for predictive models relating to SAD, given the large body of research indicating amygdala alterations in SAD and other anxiety disorders^24,25^, and with several studies reporting that features of amygdala function predict CBT response in SAD patients^1,29,30^. However, the particular predictive amygdala features have varied widely from study to study, often with little overlap. It is not enough to know that “something about the amygdala” predicts treatment response—precisely specified models are needed. An aggregation of all relevant datasets with resting-state fMRI prior to CBT in SAD will provide a strong foundation for collaborative efforts to develop predictive models. While the specific model tested here may not explain sufficient variance in independent datasets for scientific or clinical applications, we believe our results can support a continued focus on amygdala connectivity in developing improved predictive models.

There are many exciting opportunities as well as challenges in the effort toward building generalizable predictive biomarkers. We believe collaborative independent replication attempts, such as the one undertaken here, will play a critical role in this process, regardless of replication results.
